# Phase-multiplied interferometry via cavity dynamics for resolution-enhanced coherent ranging

**DOI:** 10.1038/s41377-025-02160-x

**Published:** 2026-01-12

**Authors:** Yifan Wang, Jinsong Liu, Chenxiao Lin, Xin Xu, Yu Wang, Xinhang Yang, Binbin Xie, Jibo Han, Tengfei Wu, Xuling Lin, Liangcai Cao, Hongbo Sun, Yidong Tan

**Affiliations:** 1 State Key Laboratory of Precision Measurement Technology and Instruments, Department of Precision Instruments, Tsinghua University, Beijing, China; 2 National Key Laboratory of Metrology and Calibration, Beijing Changcheng Institute of Metrology & Measurement, Beijing, China; 3 Beijing Institute of Space Mechanics and Electricity, Beijing, China

**Keywords:** Optical metrology, Imaging and sensing, Optical physics

## Abstract

Coherent light detection and ranging (LiDAR) has become an indispensable tool in autonomous systems, offering exceptional precision and ambient-light immunity. Recently, applications spanning from scientific research to advanced manufacturing have increasingly required resolution that exceeds current capabilities, which faces a fundamental trade-off between improved performance and system complexity. In this study, we overcome the intrinsic limitation and present a cavity dynamics-enabled approach that actively enhances the ranging resolution through phase multiplication. By injecting target-scattered light into the optical resonator, the operating frequency of the laser undergoes periodic modulation, generating interference harmonics that multiply the phase sensitivity. Experimentally, we observe the excitation of up to the 13th-order harmonic and effective phase multiplication without physical modulation extensions, which enables more than 10 times resolution enhancement for ranging. Owing to the intrinsic phase correlation between the fundamental wave and harmonic waves, the phase noise is effectively controlled, resulting in high-precision ranging with a standard deviation on the order of tens of micrometers. The system concurrently leverages laser feedback sensitivity, achieving significant signal-to-noise ratio (SNR) improvement. With its enhanced resolution, low photon consumption, and low-cost implementation, this technology demonstrates new capabilities that promise to enable a wide range of applications.

## Introduction

Coherent metrology, which is known for its high precision, immunity to ambient light, and traceability, represents an important class of methods with extensive applications in both scientific and industrial fields, such as space exploration, medical diagnosis, and advanced manufacturing^1–4^. In particular, the flourishing developments of automatic driving, precision fabrication, and 3-dimensional (3D) profilometry have driven the need for accurate and dynamic measurements^5–9^, which are consistent with the advantages of coherent ranging methods. Consequently, coherent ranging methods have become a focus of research, and recent advancements have significantly improved their capabilities. Chip-scale integration and parallel measurement methods increase the measurement efficiency^10–12^, high repetition-rate modulation enables local vibration monitoring^13^, and narrow-linewidth lasers increase the measurement range^14^. These innovations have expanded the applicability of coherent ranging techniques in various scenarios.

Resolution is an important metric for assessing coherent ranging systems; however, most coherent ranging methods have an intrinsic trade-off between resolution enhancement and system complexity. In frequency-modulated continuous-wave (FMCW) systems, among the most prevalent coherent ranging techniques, resolution is essentially governed by the sweep bandwidth. Employing sources with extended sweep ranges offers a direct pathway to resolution improvement. Conventional solutions such as external cavity diode lasers (ECDLs) have limitations because of mechanically constrained tuning speeds despite their terahertz-scale bandwidths^8,15^. Alternative swept sources, including vertical cavity surface emitting lasers with micro-electromechanical systems-based tuning mechanisms (MEMS-VCSELs)^16–18^, Fourier domain mode-locked lasers (FDMLs)^19,20^, time-stretching lasers^14,21,22^, and stitched-DFB arrays^23,24^, offer exceptional bandwidth performance. Their bandwidths reach tens of nanometers or greater, enabling micrometer-scale resolution at modulation frequencies near 100 kHz. Nevertheless, these approaches exhibit significant complexity in source control and signal analysis. Furthermore, multiwavelength interferometry requires multiple precisely wavelength-stabilized sources, because resolution critically relies on wavelength combinations^25,26^. Frequency-comb-based coherent ranging methods achieve high resolution. However, as reported for dual-comb-based^27–33^, repetition-rate-modulated^6^, and carrier-envelope-offset frequency-modulated^11^ approaches, stringent frequency locking in addition to extra optical amplifiers and costly instrumentation is necessary. Consequently, implementing high-resolution ranging using a simple system remains challenging.

With respect to signal analysis, the phase of the interference signal in coherent ranging allows for measurement result demodulation, and the sensitivity to phase increments determines the achievable resolution of systems^24,34^. Thus, phase manipulation, through multiplication, noise reduction, or correlation analysis, offers a pathway to resolution enhancement without solely relying on hardware extensions. Techniques such as beat signal mixing^35,36^ and deep learning-based signal prediction^37^ are promising in this regard. However, these methods have limited multiplication factors and extensive prior data requirements, which hinder real-time, high-precision applications.

In this study, we present a laser-feedback-based phase multiplication (LFPM) method for resolution-enhanced coherent ranging, which exploits nonlinear effects in cavity dynamics to multiply the interference signal phase. Laser feedback occurs when the output light partially returns to the resonator^38–40^. The reinjected light interferes with the intracavity light field, generating a spontaneously amplified beat signal^41,42^, which has been reported in our previous work^43^, and affects the cavity mode. With nonlinear cavity dynamics, the system generates harmonics of the interference signal and multiplies the phase sensitivity. Owing to the stable phase correlation between the harmonics and the fundamental wave, the phase noise in higher-order signals remains controllable, achieving tens of micrometer-level standard deviations in practically repeated measurements. Furthermore, the system inherits the high sensitivity of laser feedback, which simultaneously provides intensity amplification across various harmonic orders to facilitate effective detection. Notably, the ability to selectively employ different harmonic orders allows for dynamic trade-offs between resolution enhancement and detection sensitivity. This intrinsic flexibility renders the LFPM technique adaptable to a broad spectrum of applications, including fiber-optic sensing, target tracking, and high-precision positioning.

In addition to laser ranging, the proposed LFPM method has the potential to enhance phase sensitivity in other coherent measurement tasks, where measurements often translate to detecting phase changes in specific physical processes^44–46^. Compared with existing techniques, e.g., using multiphoton number and path-entangled (NOON) states, nonlinear optical crystals, and optical path multiplication^47,48^, our method has the distinct advantages of a low excitation threshold, high harmonic multiplicity, simple structure, and low implementation complexity, rendering it applicable to various heterodyne interferometry challenges.

## Results

### Concept of the LFPM ranging system

**Fig. 1:**
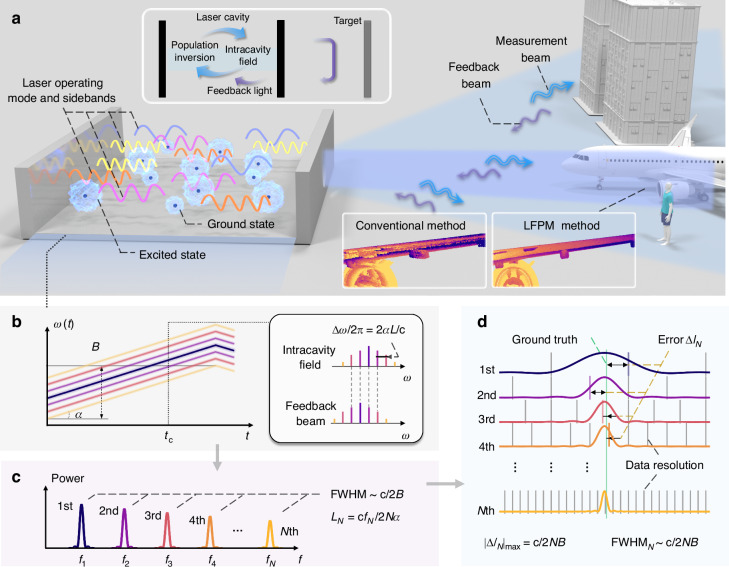
**Phase-multiplied interferometry for resolution-enhanced ranging**. **a** Concept diagram of the LFPM system. The laser emits output light directly toward the target. The feedback light re-enters the laser cavity, interferes with the intracavity field, and induces phase and intensity modulations in cavity dynamics. Top inset: schematic of the interaction between feedback light and intracavity-stimulated radiation. Bottom inset: simulated detection results using the conventional FMCW method and our LFPM method for a local area of the wing. **b** Optical frequencies of the measurement and the feedback beam under optical feedback conditions. The inset shows the comb-like spectrum of the laser at time $t={t}_{{\rm{c}}}$. $B$ represents the sweep bandwidth, $\alpha$ denotes the chirp rate, $L$ indicates the distance to be measured, and $N$ represents the order of the harmonics. **c** Harmonic generation of the interference signal originating from the mixing of the comb lines. Each harmonic has a coincident bandwidth determined by the sweep bandwidth. **d** Ranging results obtained using harmonics. Higher-order signals provide better resolution and less error

A conceptual diagram of the LFPM ranging system is shown in Fig. 1a. A tunable laser with internal modulation emits a frequency-swept beam. The measurement beam is directed toward the target, and the backscattered light is reinjected into the laser resonator. This feedback light, delayed by time $\tau$ relative to the intracavity field, generates a beat signal through coherent mixing with the local oscillator. If the optical frequency is swept linearly with time in a single trip (with the fixed chirp rate $\alpha$), the beat frequency is constant. Additionally, the feedback light participates in the stimulated emission process, periodically modulating the gain-loss parameters of the resonator at the beat frequency, resulting in dynamic modulation of the phase and intensity of the intracavity optical field.

Phase modulation manifests as the generation of new optical frequency components. Under optical feedback conditions, the laser exhibits a comb-like spectrum, as shown in Fig. 1b, where the frequency interval between the comb lines coincides with the difference between the intracavity optical field and the feedback beam. The interference between various sidebands produces the harmonics of the fundamental beat signal in Fig. 1c. Moreover, intensity modulation induced by the feedback light results in periodic fluctuations in the population inversion and gain coefficient, spontaneously amplifying the amplitude of the beat signal. Notably, when the beat frequency approaches the relaxation oscillation (RO) frequency, the typical amplification factor exceeds 10^4^. With gain saturation, the beat signal and its harmonics undergo nonlinear amplification. Overall, this laser feedback system induces nonlinear cavity dynamics and thus exhibits harmonic beat-signal generation.

The representative harmonic spectrum in the LFPM method is shown in Fig. 1c, and the full width at half maximum (FWHM) of the harmonic frequencies exhibits consistent values inversely proportional to sweep bandwidth $B$. This phenomenon enables a significant improvement in the ranging resolution when the *N*th harmonic signal is used. Because the effective sweep bandwidth is expanded by a factor of *N*, the beat frequency of the *N*th harmonic is correspondingly *N* times greater than that of the fundamental signal. Consequently, the raw distance obtained from the *N*th harmonic must be divided by *N* to yield the actual target distance. Moreover, the FWHM is reduced to 1/*N* of its fundamental value. This narrower spectral linewidth indicates a finer effective frequency sampling interval. As a result, the detrimental fence-posting effect intrinsic in the discrete Fourier transform is mitigated, reducing uncertainty in locating the true peak frequency. Ultimately, this decreased peak frequency estimation error translates directly to a lower ranging error relative to the ground truth, increasing both the resolution and precision (Fig. 1d).

### Principle of phase multiplication

**Fig. 2:**
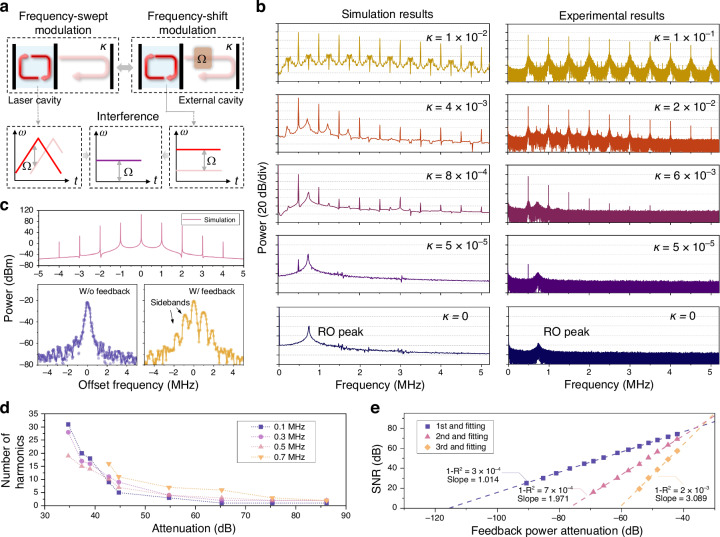
**Principles of phase multiplication via laser feedback**. **a** Schematic of the frequency-swept modulation and frequency-shifted modulation systems. In both configurations, a stable frequency difference exists between the feedback light and the intracavity field. Consequently, their cavity dynamics with laser feedback are similar. **b** Simulation results based on the L‒K equations and experimental measurements at the fixed frequency shift $\varOmega /2{\rm{\pi }}=500$ kHz. The number of harmonics progressively increases with increasing feedback strength $\kappa$. **c** Top: simulated laser output spectrum. The frequency interval between sidebands corresponds to the set modulation frequency $\varOmega /2{\rm{\pi }}=1$ MHz. Bottom: experimentally measured laser output spectrum. Modulation sidebands emerge under optical feedback, aligning with the simulation results. **d** Number of harmonics in interference signals excited at a fixed $\kappa$ for different modulation frequencies. **e** SNR of interference signal harmonics versus feedback power attenuation (${\kappa }^{2}$). Linear relationships are observed for the harmonics

Higher-order harmonic generation originates from the nonlinear cavity dynamics induced by laser feedback and can be described within the semiclassical framework using rate equations. In conventional Lang–Kobayashi (L‒K) feedback models^49,50^, the phase difference between intracavity light and feedback light is the focus. To elucidate the underlying mechanism, we analyze the feedback process in a free-running source under frequency-shifted modulation to investigate that of a frequency-swept laser source, where the frequency shift corresponds to the beat frequency generated in the ranging signal, as shown in Fig. 2a. This approximation remains valid given that the cavity mode sweeping rate is substantially lower than the laser buildup rate. $N\left(t\right)$, ${E}_{{\rm{c}}}\left(t\right)$, and $\varPhi \left(t\right)$ denote the population inversion, the complex amplitude of the electric field in reduced units (photon units), and the optical phase, respectively. The rate equations with laser optical feedback are expressed as follows:

1 $$\frac{{dN}\left(t\right)}{{dt}}={\gamma }_{1}\left[{N}_{0}-N\left(t\right)\right]-{B}_{{\rm{E}}}N\left(t\right){\left|{E}_{{\rm{c}}}\left(t\right)\right|}^{2}$$

2 $$\frac{d{E}_{{\rm{c}}}\left(t\right){e}^{i\varPhi \left(t\right)}}{{dt}}=\left[i{\omega }_{{\rm{c}}}+\frac{{B}_{{\rm{E}}}N\left(t\right)-{\gamma }_{{\rm{c}}}}{2}\right]{E}_{{\rm{c}}}\left(t\right){e}^{i\varPhi \left(t\right)}+\kappa {\gamma }_{{\rm{c}}}{E}_{{\rm{c}}}\left(t-\tau \right){e}^{i\varPhi \left(t-\tau \right)}{e}^{i\Omega t}$$

where ${\gamma }_{1}$ represents the decay rate of the population inversion; ${\gamma }_{1}{N}_{0}$ represents the pumping rate; ${B}_{{\rm{E}}}$ denotes the Einstein coefficient; ${\omega }_{{\rm{c}}}$ indicates the laser cavity frequency; ${\gamma }_{{\rm{c}}}$ represents the decay rate of the photon inside the cavity; $\kappa$ denotes the feedback strength, which is defined as the square root of the feedback-to-output power ratio; $\tau$ indicates the transit time of the photon in the external cavity; and $\varOmega$ represents the round-trip frequency shift of the light provided by the frequency shifter. For simplicity, we assume that the round-trip time outside the cavity is shorter than the period of the frequency-shifted modulation ($\varOmega \tau \ll 1$), and ${E}_{{\rm{c}}}\left(t-\tau \right)\approx {E}_{{\rm{c}}}\left(t\right)$. The evolution of ${E}_{{\rm{c}}}\left(t\right)$ can be simplified as follows:

3 $$\frac{d{E}_{{\rm{c}}}\left(t\right)}{{dt}}=\frac{{B}_{{\rm{E}}}N\left(t\right)-{\gamma }_{{\rm{c}}}}{2}{E}_{{\rm{c}}}\left(t\right)+\kappa {\gamma }_{{\rm{c}}}{E}_{{\rm{c}}}\left(t\right)\cos \left[\varOmega t-\omega \left(t\right)\tau \right]$$

4 $$\omega \left(t\right)={\omega }_{{\rm{c}}}+\kappa {\gamma }_{{\rm{c}}}\sin \left[\varOmega t-\omega \left(t\right)\tau \right]$$

Consequently, the running laser frequency $\omega \left(t\right)$ is modulated and fluctuates regularly: the magnitude correlates with the feedback strength $\kappa$, and the period matches the modulation frequency $\varOmega$. Equation (4) reveals that the laser generates multiple modulation sidebands. Interference occurs between these sidebands, thereby generating harmonic beat signals, with frequencies $\varOmega ,2\varOmega ,3\varOmega$, …, and corresponding multiple phase shifts.

On the other hand, the harmonic signal can be amplified spontaneously by cavity dynamics. Through linear perturbation analysis of Eqs. (1) and (3), the relative variation in the population inversion Δ*n(t)* can be derived as follows:

5 $$\Delta n\left(t\right)\propto \frac{\kappa {\gamma }_{{\rm{c}}}{\gamma }_{1}\left(\eta -1\right)}{\sqrt{{\left[{\gamma }_{{\rm{c}}}{\gamma }_{1}\left(\eta -1\right)-{\varOmega }^{2}\right]}^{2}+{\left({\gamma }_{1}\eta \varOmega \right)}^{2}}}\cos \left(\varOmega t-{\omega }_{{\rm{c}}}\tau \right)$$

where $\eta ={B}_{{\rm{E}}}{N}_{0}/{\gamma }_{{\rm{c}}}$ represents the normalized pumping rate. The fluctuations of population inversion, as well as the gain saturation effect, periodically modulate the optical gain and thus affect the intensity of the harmonics. $\Delta {i}_{N}$ represents the relative intensity modulation of the *N*th harmonic, and ${G}_{N}$ represents the gain factor.

6 $$\Delta {i}_{N}\propto {\kappa }^{N}{G}_{N}\left(N\varOmega \right)\cos \left(N\varOmega t-N{\omega }_{{\rm{c}}}\tau \right)$$

Numerical simulations are performed, and the modulation frequency is 500 kHz. The numerical solution of Eq. (4) is obtained and then substituted into Eq. (3). The evolution of the normalized power spectrum of the laser output with various $\kappa$ values is shown in Fig. 2b. With respect to $\kappa =0$ (free-running), a single RO peak is observed at 750 kHz. As the feedback strength $\kappa$ increases, a beat frequency signal emerges, after which harmonic signals appear. When $\kappa$ exceeds 10^-3^, the number of harmonics progressively increases, up to the 10th.

Experimental validation is conducted. An acousto-optic modulator is employed to induce a frequency shift for the feedback light. We use an adjustable attenuator to control the feedback power. The evolution of the output spectrum at various values of $\kappa$ is in agreement with the simulations. Minor discrepancies in absolute feedback strength values may arise from mode mismatches (e.g., polarization or transverse modes) between the feedback light and the intracavity field. The fine laser spectra from the simulations and experiments are shown in Fig. 2c, revealing a comb-like structure with a comb-line spacing equal to the modulation frequency. The setup is shown in Supplementary Information 1. We also record the number of harmonics at other modulation frequencies. The harmonic counts corresponding to $\varOmega /2{\rm{\pi }}$ values of 100 kHz, 300 kHz, 500 kHz, and 700 kHz tend to decrease with increasing attenuation, as shown in Fig. 2d.

To further investigate the relationship between the feedback strength and the harmonic intensity response, we attenuate the return light to measure the signal-to-noise ratio (SNR). The attenuation curves for the 1st, 2nd, and 3rd harmonics exhibit linear responses on a logarithmic scale, as presented in Fig. 2e, and the corresponding slopes of the linear fits are 1.01, 1.97, and 3.09, respectively. This observation reveals that the intensity of the *N*th harmonic scales with the *N*th power of the feedback level. Higher-order harmonics are more sensitive to attenuation of the feedback light, which is consistent with Eq. (6). These experimental results are consistent with the simulation results, confirming the validity of our theoretical model. Notably, PD noise also affects the SNR and the number of detectable harmonics. Further details are available in Supplementary Information 2.

### Ranging results with enhanced resolution

**Fig. 3:**
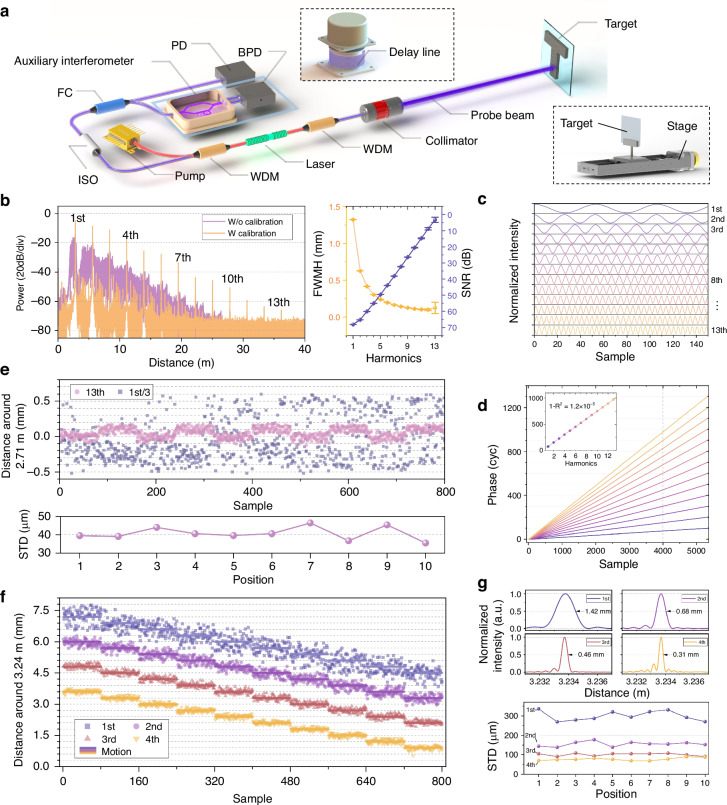
**Ranging performance of LFPM**. **a** Schematic of the LFPM systems. WDM represents wavelength division multiplexer, ISO denotes optical isolator, FC indicates fiber coupler, PD represents photodetector, and BPD denotes balanced photodetector. Top inset: packaged delay line of the auxiliary interferometer. Right inset: the target for the resolution tests. **b** Power spectra with and without frequency nonlinearity calibration when a reflector is used as the target. Harmonics up to the 13th are excited. Right: the SNRs and FWHMs of the harmonics. Higher-order phase multiplication results in lower SNR. **c** Temporal signals of the harmonics. **d** Phase increments of the harmonics. The inset shows the linear relationship between the unwrapped phase and the harmonics. **e** Experimental results for a shiny target. The target undergoes a reciprocating motion in steps of 0.1 mm. Top: ranging results. For clarity, the offset ranging results of the fundamental wave are scaled down by a factor of 3. Bottom: standard deviation of 80 measurements at 10 corresponding positions. STD represents standard deviation. **f** Experimental results for noncooperative targets. Higher-order harmonic waves exhibit higher resolution. **g** Top: the FWHM of the 1st to 4th harmonics. Bottom: the STD of 80 measurements at 10 corresponding positions

We perform absolute distance measurement experiments on the basis of our system and evaluate the performance of phase multiplication. The experimental setup is shown in Fig. 3a. The output power of the laser source is nearly 2 mW, with a maximum sweep bandwidth of 110 GHz. The main power serves as the measurement beam for ranging, whereas the minor power from the other port is split further for detection and calibration. We set up an auxiliary interferometer to calibrate the swept-frequency nonlinearity^51^, which is critical for high-precision distance measurement (explained in the Materials and Methods section), and the entire packaged device is monitored by a temperature controller. A photodetector (PD) is used to receive the beam from the laser. Because the beat signal is generated within the resonant cavity, directly analyzing the output light of the laser can yield the measured distance.

The ranging results when a reflector is employed as the target are presented in Fig. 3b. With the auxiliary interferometer, nonlinear phase noise is suppressed, and harmonics up to the 13th order in the calibrated output spectrum are clearly observed. In contrast, prior to this correction, adjacent harmonics are indistinguishable because of severe spectral broadening. Both the SNR and the FWHM values of the calibrated harmonics decrease with increasing harmonic order. We separate the original interference signals during a chirp process with bandpass filters and obtain the harmonic components in the time domain. The normalized temporal signals are shown in Fig. 3c, and the Hilbert transform of these signals reveals their phase evolution in Fig. 3d. The inset, which highlights the phase increments of different harmonics at an identical time point, shows a distinct linear relationship. These observations are in agreement with our theoretical analysis and validate the capability of phase multiplication of our proposed method.

Resolution tests of the LFPM are conducted. A shiny target is displaced by 0.1 mm using a translation stage, with 80 ranging measurements obtained at each position. The fundamental frequency corresponds to a target distance of 2.71 m. As shown in Fig. 3e, the results of the 13th harmonic clearly reconstruct the motion, and the standard deviation at each position is less than 50 μm, while the results of the fundamental wave cannot be resolved. For noncooperative targets, results employing harmonics are obtained from an aluminum block at 3.24 m with an effective reflectivity of 2 × 10^−6^, which is far from the mirror reflectivity. A weak feedback strength results in a decrease in the number of excited harmonics, as shown in Fig. 2e, and up to the 4th harmonic is observed in the experiments. The displacement is 0.3 mm in each step. As shown in Fig. 3f, higher harmonics progressively reconstruct the motion trajectory. The intensity-normalized interpolated spectra of the 1st to 4th harmonics, as shown in Fig. 3g, reveal a distinct decrease in the FWHM from 1.4 mm to 0.3 mm. The precision analysis, evaluated through 80 repeated measurements, reveals a standard deviation of 76 μm for the 4th harmonic, corresponding to a fourfold improvement in measurement precision compared with the fundamental wave with 300 μm.

### LiDAR and 3D imaging

**Fig. 4:**
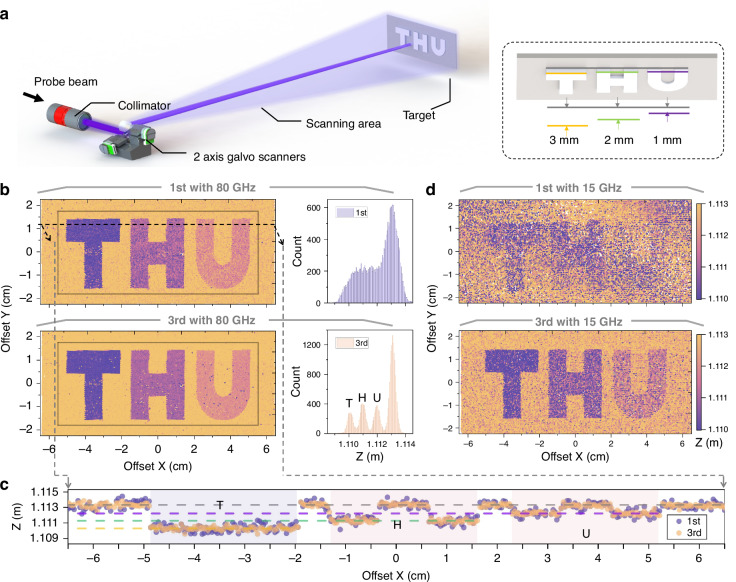
**3D imaging results**. **a** Experimental setup for beam scanning and target detection. Inset: thicknesses of three aluminum blocks. **b** Reconstructed images obtained with a sweep bandwidth $B=80$ GHz, which is based on fundamental interference signals and the 3rd harmonic signals. Right: height distribution of the point cloud within the gray rectangle. **c** Measured height variation along the black dashed line in b, which shows block and background regions and clearly distinguishes the three blocks from the background in the 3rd results. **d** Imaging results obtained with $B=15$ GHz

The LFPM method simultaneously achieves substantial resolution enhancement and reduces the implementation complexity, revealing its potential for 3D imaging. We experimentally validate this concept using a two-axis scanning galvanometer to steer the light beam across horizontal and vertical coordinates (Fig. 4a). Three aluminum blocks (with thicknesses of 3, 2, and 1 mm along the axis) forming the letters “THU” are positioned 1.1 m from the collimator as targets. A schematic of the data acquisition is provided in Supplementary Information 3. We propose an adaptive intensity compensation method using a semiconductor optical amplifier (SOA) to compensate for the feedback power fluctuations, and the system and performance are elucidated in Supplementary Information 4.

The results obtained with an effective sweep bandwidth of 80 GHz are presented in Fig. 4b. Fourier analysis is directly applied to the calibrated beat signals without frequency-domain interpolation. After the resolution enhancement of the LFPM, the 3rd harmonic reconstructions exhibit superior fidelity compared with those of the fundamental signal. As indicated by the dashed horizontal slice in Fig. 4c, the 3rd harmonic profile shows smoother outlines with reduced noise, closely aligning with the ground truth. The deviation of each individual target is less than 200 μm, which originates from optical path fluctuations induced by the shaking of the galvanometers. Data distribution statistics are also performed to quantify the degree of concentration of the results, as shown in the histograms in Fig. 4b. The separability of the background and the three targets is significantly improved through the proposed LFPM method. The sweep bandwidth is further reduced to 15 GHz, a range achievable with conventional phase modulators. Under this condition, the measurements based on both the conventional method and the proposed LFPM method are shown in Fig. 4d. The reconstruction with the fundamental wave is difficult to resolve, whereas that with the 3rd harmonic can be clearly recognized.

## Discussion

Our work establishes a technique for resolution-enhanced laser ranging. By leveraging the laser feedback effect, backscattered light interferes with the intracavity field. This process actively generates the harmonics of the beat signal through nonlinear cavity dynamics, including cavity-mode frequency modulation and gain saturation. Note that the harmonics are not spurious artifacts. Instead, they intrinsically carry phase information that is multiplicatively enhanced to increase the ranging resolution and precision. Consequently, the laser cavity itself functions not only as a signal generator but also as an active nonlinear phase-processing element. This functionality is achieved without the need for complex external optical modulators or operating at high energy thresholds. In the experiments, the μW-level feedback power generates harmonics exceeding the 10th order, and we achieve 3-fold to 13-fold phase multiplication. In 3D imaging, our method resolves objects spaced 1 mm apart using only a 15 GHz sweep bandwidth without interpolation. Additionally, the LFPM method has a ranging capacity that is greater than 100 m, with a standard deviation on the order of tens of micrometers (Supplementary Information 5).

**Fig. 5:**
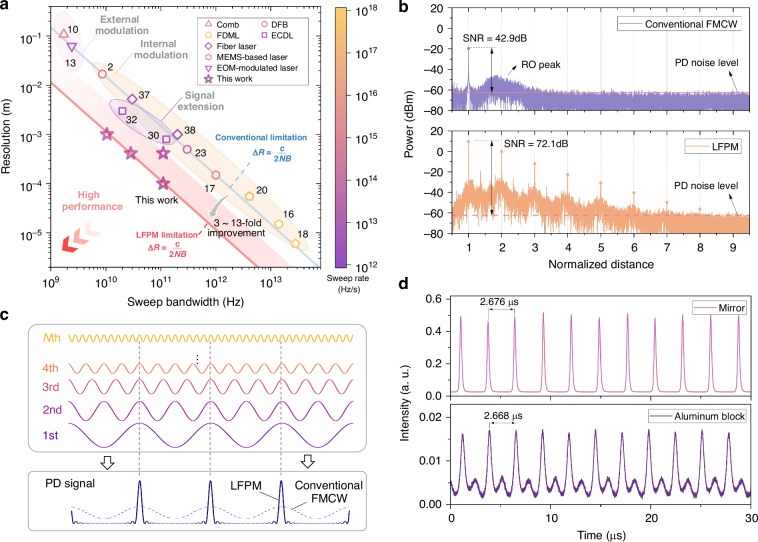
**Comparison with conventional FMCW methods**. **a** Performance in comparison with other reports. The blue solid line represents the intrinsic limitation in FMCW systems between the sweep bandwidth $B$ and resolution $\Delta R$, whereas the red zone represents the LFPM with the harmonics from the 3rd to 13th orders. Higher resolutions with smaller bandwidths are desirable. The color scale represents the chirp rate across different studies. **b** Power spectra of the conventional FMCW and LFPM under identical experimental conditions. The dashed lines indicate the PD noise level. The distance has been normalized to the fundamental peak. **c** Temporal performance of the LFPM system. Harmonics possess multiple phases with coherence, and their superposed signal exhibits sharper peaks than those of the conventional method, which uses the fundamental wave. **d** Experimentally detected signals in the LFPM system targeting a mirror and an aluminum block. The shape of the peaks relies on the number of generated harmonics. These sharp peaks enable the acquisition of the beat frequency via the time interval between adjacent peaks, yielding a high acquisition rate

We compare the performance of our proposed LFPM method with that of the representative reported FMCW ranging methods in Fig. 5a. The proposed LFPM method overcomes the intrinsic limitation in frequency-domain interferometry between the resolution and sweep bandwidth and improves it toward a more desirable level. With the generation of higher-order harmonics, the performance can be improved further. With respect to phase, this harmonic-induced multiplication is equivalent to extending the effective sweep range of the system, which prevents the resolution from being limited by the physical bandwidth. This mechanism leverages the intrinsic nonlinear dynamics of the laser cavity rather than circumventing basic Fourier analysis. Compared with parallel coherent ranging systems, such as dual-comb ranging and multiwavelength interferometry, the LFPM method has a compact size and low photon consumption. Compared with NOON-state-based phase multiplication techniques, our system demonstrates a higher enhancement factor and is robust under ambient noise and vibrational interference. Furthermore, intracavity interference eliminates the need for external reference paths in our LFPM system, enabling a compact design with reduced complexity. This optimized approach has vast potential for scientific and industrial applications, including remote target tracking and positioning.

To clearly validate the generation of harmonics originating from laser cavity dynamics instead of electrical factors, we perform a comparison with a conventional FMCW-based ranging system (Supplementary Information 6). The conventional system exhibits no generation of harmonics, as shown in Fig. 5b. Moreover, the intensity response of the fundamental signal also decreases, whereas the SNR of the LFPM is nearly 30 dB greater. This distinction arises from the noise mechanisms. In conventional FMCW systems, all noise sources, particularly PD noise and laser intensity noise, decrease the SNR. Conversely, the LFPM leverages intracavity interference to render the SNR partially independent of PD noise. Optical feedback thus acts as an effective PD-noise suppressor, significantly improving signal detectability. This improvement indicates that the proposed system is advantageous in both phase and intensity sensitivity. Notably, laser feedback achieves phase multiplication not limited to the ranging system, and it enables enhanced resolution with weak return power in other interferometric systems, such as heterodyne interferometry for displacement measurements.

Despite the advantages mentioned above, some aspects can still be improved in the LFPM system in the future. First, the laser-feedback-induced intensity amplification for the harmonics is not flat, and the response frequency bandwidth is limited by the laser dynamics (Supplementary Information 7). The gain of the amplification is associated with the frequency of the beat signal, and the maximum is achieved when the beat frequency equals the RO frequency. The SNR and sensitivity of higher-order harmonics can be further increased through appropriate control of the self-mixing gain by matching the frequency, such as adjusting the RO frequency or adding frequency-shifted devices. Moreover, the feasible dynamic range for the feedback strength is relatively small, especially for high-order harmonics, because of the power relationship of intensity degradation. On the other hand, the feedback strength cannot increase indefinitely, and single-mode operating conditions should be guaranteed. Strong optical feedback results in multiple emission frequencies, bifurcation, mode hopping, or even chaos^52^. Consequently, there is a trade-off between the resolution enhancement and detection sensitivity, and the fundamental wave has the highest detection sensitivity. The LFPM system allows for the selection of an appropriate harmonic order to match scenarios with different resolution and sensitivity requirements, supporting greater applicability across diverse measurement tasks.

In terms of other factors affecting performance, although this work emphasizes a novel mechanism for resolution enhancement, the effective sweep bandwidth of the LFPM, while in the THz range, remains lower than that of typical FDML or VCSEL-based FMCW systems. In our system, the available effective bandwidth relies on the maximum variation in the laser cavity length, which is achieved by mechanically stretching the whole fiber laser. To achieve higher resolution rather than merely enhancing it by phase multiplying, the selection of a more powerful actuator or other devices with an adjustable cavity becomes essential. Notably, spectral interpolation of the interference signal, such as zero-padding interpolation or chirp-Z transform, is a common method for improving data resolution. However, these methods often result in a substantial increase in computational complexity, including a higher sampling rate and a larger amount of data in Fourier analysis, which makes it difficult for them to meet the requirements of real-time measurement scenarios. We also evaluate the performance of the LFPM method using frequency-domain interpolation, and detailed results are provided in Supplementary Information 8. The measurement update rate of the current system is constrained to tens of hertz by its mechanical modulation scheme, which also limits the achievable chirp rate. However, this limitation is not intrinsic to the LFPM principle itself, which is directly applicable to other laser platforms with compatible cavity dynamics, such as laser diodes and solid-state microchip lasers. Faster tuning methods can be adopted according to their own characteristics. In future work, we will focus on the development of new light sources to achieve fast frequency-swept modulation and high update-rate measurements.

Finally, it is noteworthy that the proposed LFPM method can also provide the potential to replace the complex analysis in the frequency domain, which is typically required in conventional FMCW, with that in the time domain. The cavity dynamics of the laser feedback system induce the spontaneous generation of the harmonics of the beat signal. Importantly, these harmonics exhibit stable integer‒multiple phase relationships. The detector receives the superposition of these signals. Like mode-locked signals, the detected signals exhibit sharper signal peaks in the temporal interferogram, as shown in Fig. 5c. The time intervals between these peaks directly correspond to the inverse of the beat frequency, enabling high-speed acquisition for ranging. The experimental signals are shown in Fig. 5d, targeting a mirror and an aluminum sheet. This is similar to a reported ranging system based on multi-sideband swept modulation^6^. However, our approach achieves effective bandwidth extension instead of physically broadening the sweep range and, critically, does not require expensive external modulation devices. Additionally, in our LFPM method, it is necessary to calibrate frequency nonlinearities intrinsic to the cavity mode sweeping, such as using pre-correction techniques^53^.

## Materials and methods

### Analysis of laser running frequency

According to Eqs. (1) and (2), the evolution of ${E}_{{\rm{c}}}\left(t\right)$ and $\varPhi \left(t\right)$ can be obtained by separating the real and imaginary parts^52^.

7 $$\frac{d{E}_{{\rm{c}}}\left(t\right)}{{dt}}=\frac{{B}_{{\rm{E}}}N\left(t\right)-{\gamma }_{{\rm{c}}}}{2}{E}_{{\rm{c}}}\left(t\right)+\kappa {\gamma }_{{\rm{c}}}{E}_{{\rm{c}}}\left(t-\tau \right)\cos \left[\varOmega t+\varPhi \left(t-\tau \right)-\varPhi \left(t\right)\right]$$

8 $$\frac{d\varPhi \left(t\right)}{{dt}}={\omega }_{{\rm{c}}}+\kappa {\gamma }_{{\rm{c}}}\frac{{E}_{{\rm{c}}}\left(t-\tau \right)}{{E}_{{\rm{c}}}\left(t\right)}\sin \left[\varOmega t+\varPhi \left(t-\tau \right)-\varPhi \left(t\right)\right]$$

where $d\varPhi \left(t\right)/{dt}$ represents the optical running laser frequency $\omega \left(t\right)$. $\varPhi (t-\tau )$ can be expanded in terms of time delay $\tau$ using the Taylor series. When $\tau$ is much smaller than $1/\varOmega$, higher-order items except the linear term can be neglected, $\varPhi \left(t-\tau \right)-\varPhi \left(t\right)\approx -\omega \left(t\right)\tau$, and the electric field fluctuations can also be neglected, ${E}_{{\rm{c}}}\left(t-\tau \right)\approx {E}_{{\rm{c}}}\left(t\right)$. Furthermore, Eq. (8) can be simplified to Eq. (4). When the feedback strength satisfies $\kappa {\gamma }_{{\rm{c}}}\tau < 1$, the single-mode condition, this equation yields a unique solution. The value range of the solution scales proportionally with the feedback strength and exhibits periodic oscillations. The specific analysis is described in Supplementary Information 9. To simplify the mathematical expression, the phase shift induced by frequency fluctuations can be neglected further, and the output optical field of the laser can be described by Eq. (9). Expansion using Bessel functions reveals modulation sidebands in the optical spectrum adjacent to the intrinsic mode.

9 $$\Delta {E}_{c}\left(t\right)\propto \cos \left[{\omega }_{c}t+\kappa {\gamma }_{c}/\varOmega \cos \left(\varOmega t-{\omega }_{c}\tau \right)\right]\propto \mathop{\sum }\limits_{N\in Z}^{\infty }{J}_{N}\left\{\cos \left[\left({\omega }_{c}+N\varOmega \right)t-N{\omega }_{c}\tau \right]\right\}$$

### Characteristics of the laser source

The laser source employed is an erbium-ytterbium co-doped distributed feedback (DFB) fiber laser, characterized by its compact architecture, prominent RO characteristics, and wide mode-hop-free tuning range. The DFB laser is pumped by a 976 nm laser diode and emits dual-port beams centered at 1545 nm. Output beams with powers of 1.2 mW and 0.3 mW are allocated for measurement and signal analysis, respectively. An ISO is integrated into the non-probe port of the laser to suppress back-reflections from auxiliary interferometer components or photodetectors, thereby eliminating parasitic spectral signals. The free-running laser has an intrinsic linewidth of 26.4 kHz (Supplementary Information 10), as indicated by delayed self-heterodyne interferometry. Frequency-swept modulation is achieved by stretching the fiber laser cavity using a piezoelectric ceramic actuator (PZT). In the experiments, an 80 Hz symmetric triangular waveform applied to the PZT yields a sweep range of 100 GHz. A comprehensive characterization of the laser source, PZT modulation repeatability, and tuning performance is provided in Supplementary Information 11–12.

### Nonlinearity calibration and distance calculation

Owing to the nonlinearity of frequency sweeping in practice, the beat frequency during a scanning ramp fluctuates with time. In this work, an unbalanced Mach–Zehnder interferometer (MZI) is used as an auxiliary interferometer to generate a resampling clock signal for nonlinearity calibration. The normalized measurement signal ${I}_{{\rm{m}}}$ and auxiliary signal ${I}_{{\rm{a}}}$ can be denoted as follows:

10 $$\begin{array}{c}{I}_{{\rm{m}},i}\left(t\right)=\cos \left[{\varPhi }_{i}\left(t\right)-{\varPhi }_{i}\left(t-{\tau }_{{\rm{m}}}\right)\right]\\ {I}_{{\rm{a}},i}\left(t\right)=\cos \left[{\varPhi }_{i}\left(t\right)-{\varPhi }_{i}\left(t-{\tau }_{{\rm{a}}}\right)\right]\end{array}i={\rm{up}},{\rm{down}}$$

where ${\tau }_{{\rm{m}}}$ and ${\tau }_{{\rm{a}}}$ represent the time delay originating from the distance to be measured and the difference in the optical path length of the MZI, ${L}_{{\rm{aux}}}$. The subscript *i* represents the parameters in upward or downward scanning. The phase component can be expanded using a Taylor series, where higher-order terms can be neglected when

11 $$|{\tau }_{\rm{a}}^{2}\alpha |\ll 1$$

where $\alpha =dv/{dt}$ is the chirp rate. The auxiliary signal is employed to generate a sampling clock *t*_*k*_ with a phase interval of π, which is expressed as follows:

12 $$2\pi \alpha {t}_{k}{\tau }_{{\rm{a}}}=k\pi ,k\epsilon {\rm{{\rm Z}}}$$

The measurement signal is sampled, yielding

13 $${I}_{{\rm{m}},i}\left(k\right)=\cos \left(2\pi \frac{{\tau }_{{\rm{m}}}}{2{\tau }_{{\rm{a}}}}k\right)$$

The resampled signal in the k-domain is analyzed through the fast Fourier transform (FFT), and the distance to be measured is revised as follows:

14 $${L}_{N,i}=\frac{{m}_{N,i}}{n{M}_{i}N}{L}_{{\rm{aux}}}$$

where $M$ represents the length of the data after resampling for the FFT, $n$ denotes the refractive index of the medium in the measurement path delay, and ${m}_{N}$ indicates the peak position of the *N*th harmonic in the spectrum. We use the average distance from the upward and downward scans as the ranging result to compensate for the Doppler effect of ambient disturbances. Notably, in the LFPM ranging system, cavity dynamics induce extra nonlinear frequency fluctuations according to Eq. (4). However, the resampling method remains effective for the applications in this paper. The detailed derivation is provided in the Supplementary Information 13–14.

Additionally, the theoretical upper limit of the measurement range is determined by the optical path difference ${L}_{{\rm{aux}}}$. In accordance with the Nyquist sampling criterion, the maximum measurement range for the fundamental beat signal is represented as ${L}_{{\rm{aux}}}/2$. For the *N*th harmonic, the required sampling frequency increases by a factor of N. Consequently, the corresponding maximum measurement range is reduced to ${L}_{{\rm{aux}}}/2N$.

## Supplementary information

Supplementary Information for Phase-Multiplied Interferometry via Cavity Dynamics for Resolution-Enhanced Coherent Ranging

## Data Availability

The data that support the findings of this study are available from the corresponding authors upon reasonable request.
