# Supplementary material for: Phase-multiplied interferometry via cavity dynamics for resolution-enhanced coherent ranging
Source: Light Sci Appl. 2026 Jan 12;15:67. doi: 10.1038/s41377-025-02160-x (PMC12791145; doi:10.1038/s41377-025-02160-x)
Supplement: Supplementary file 1 — Supplementary Information for Phase-Multiplied Interferometry via Cavity Dynamics for Resolution-Enhanced Coherent Ranging [file 41377_2025_2160_MOESM1_ESM.pdf]

Supplementary Information for

Phase-Multiplied Interferometry via Cavity Dynamics for Resolution-Enhanced Coherent Ranging

Yifan Wang[1], Jinsong Liu[1], Chenxiao Lin[1], Xin Xu[1], Yu Wang[1], Xinhang Yang[1], Binbin Xie[1], Jibo Han[2], Tengfei Wu[2], Xuling Lin[3], Liangcai Cao[1], Hongbo Sun[1], Yidong Tan[1]

Correspondence: Yidong Tan (Tanyd@tsinghua.edu.cn)

[1]State Key Laboratory of Precision Measurement Technology and Instruments, Department of Precision Instruments, Tsinghua University, Beijing 100084, China

[2]National Key Laboratory of Metrology and Calibration, Beijing Changcheng Institute of Metrology & Measurement, Beijing 100095, China

[3]Beijing Institute of Space Mechanics and Electricity, Beijing 100094, China

These authors contributed equally: Yifan Wang, Jinsong Liu, and Chenxiao Lin

1 Optical spectrum analysis with laser feedback

With laser feedback, the output spectrum of the laser exhibits sidebands, as analyzed by Eq. (4) in the main text. The modulation frequency in this study is at the MHz level, which is significantly lower than the resolution of typical optical spectrum analyzers and scanning Fabry-Perot interferometers. Thus, it is not feasible to use conventional instruments to directly observe these sidebands[1]. To address this limitation, we construct an optical setup as shown in Fig. S1, which combines part of the output from the fiber laser with a tunable laser source (TLS) and detects the beat signal between them. The signal power spectrum is then analyzed using an electronic spectrum analyzer (ESA). The TLS used in this experiment possesses a linewidth better than 200 kHz, and its wavelength setting resolution is 0.1 pm, which allows it to match the fiber laser and ensures that the beat frequency signal falls within the detection range of the photodetector (PD). The fiber laser operates under thermal and vibration isolation conditions, and its output is frequency-shifted by a pair of acousto-optic modulators (AOMs) before returning to the laser cavity. Considering the linewidths of both light sources, the AOM modulation frequency is set to 1 MHz to effectively distinguish the sidebands. Additionally, an optical isolator (ISO) is used to eliminate the influence of the TLS on the fiber laser. We record the power spectra in the presence of frequency-shifted feedback and under free-running conditions, as shown in Fig. 2(c) in the main text.

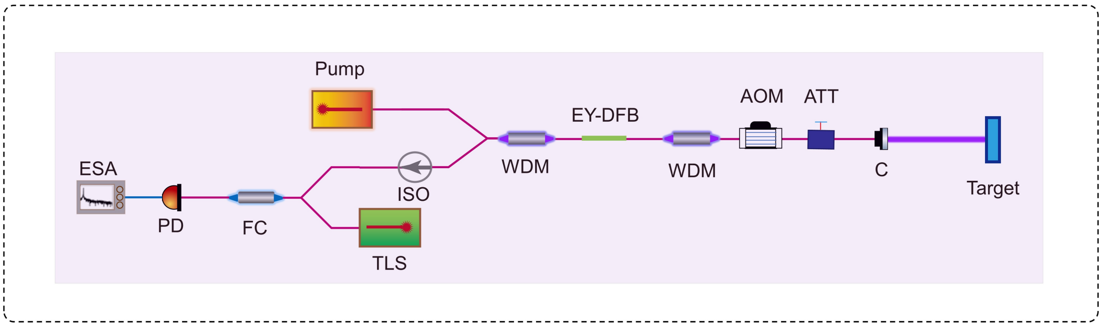

Fig. S1 Schematic diagram of fine spectrum analysis. EY-DFB, erbium-ytterbium co-doped distributed feedback laser. AOM, acousto-optic modulator. WDM, wavelength division multiplexer. ISO, optical isolator. PD, photodetector. ATT, adjustable attenuator. C, collimator. FC, fiber coupler. TLS, tunable laser source. ESA, electronic spectrum analyzer.

2 Dependence of the number of detectable harmonics on photodetector noise

The maximum detectable number of harmonics is ultimately determined by the SNR, which is susceptible to the system noise level. In a laser feedback system, the predominant noise sources are laser intensity noise and PD noise, as reported in our previous work[2]. Within the frequency band dominated by laser intensity noise, typically near the relaxation oscillation (RO) peak, the signal-to-noise ratio (SNR) of the beat signal is nearly immune to PD noise. Beyond this region, however, PD noise becomes a limiting factor, progressively degrading the SNR. Since higher-order harmonics in the LFPM system generally exhibit lower power and are located at higher frequencies, they are more vulnerable to such PD noise, thereby influencing the practically detectable harmonic number.

To experimentally validate this effect, we perform experiments in both the frequency-shift feedback system and the LFPM ranging system. In the frequency-shift configuration with $\Omega/2\pi$ = 500 kHz, power spectra obtained under different PD noise levels are shown in Fig. S2a. When the noise-equivalent power (NEP) is 68.6 pW $Hz^{-1/2}$, harmonics up to the 6th order are detectable. In contrast, with a lower NEP of 14.9 pW $Hz^{-1/2}$, the 8th harmonic becomes clearly detectable. Similar trends are observed in the LFPM ranging experiments (Fig. S2b), where the use of a low-NEP PD enables the detection of the 9th harmonic, higher than that of a high-NEP PD.

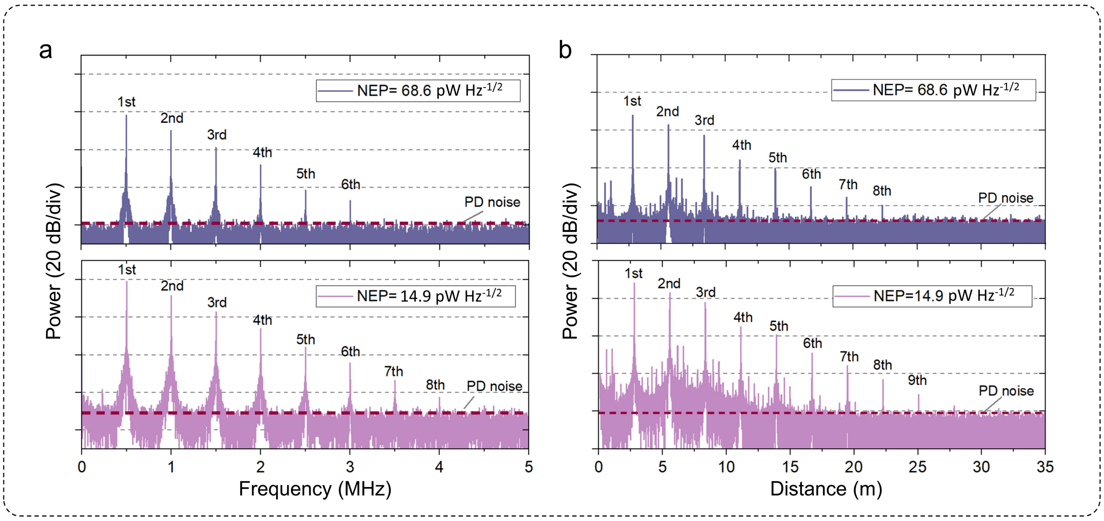

Fig. S2 The number of harmonics excited at a fixed feedback strength under different PD noise levels. a Spectra obtained in the frequency-shift modulation system. **b** Spectra obtained in the LFPM ranging system. Top: acquired with a PD of NEP = 68.6 pW $Hz^{-1/2}$. Bottom: acquired with a PD of NEP = 14.9 pW $Hz^{-1/2}$.

3 Signal generation and acquisition

The layout for signal generation and acquisition is illustrated in Fig. S3. A symmetric ramp signal generated by an arbitrary waveform generator (AWG) is amplified by a power amplifier (PA) to drive the piezoelectric ceramic actuator (PZT). Synchronization between data acquisition and galvanometer motion is ensured by a TTL trigger signal output simultaneously with the ramp signal from the AWG. Signals from the PD (corresponding to the laser output) and balanced photodetector (BPD, representing the auxiliary interferometer output) are recorded concurrently using a data acquisition card (DAQ). As theoretically predicted, the laser output contains the target beat signal for measurement, yet has no impact on the frequency nonlinearity calibration (Supplementary Information 14). The two galvanometers, controlled individually by a dual-channel galvanometer controller, remain stationary during data acquisition. Finally, recorded signals are analyzed computationally. The SNR analysis of the fundamental beat signal yields an error signal, which is output to an analog output device (AOD) for controlling the semiconductor optical amplifier (SOA) in adaptive intensity compensation.

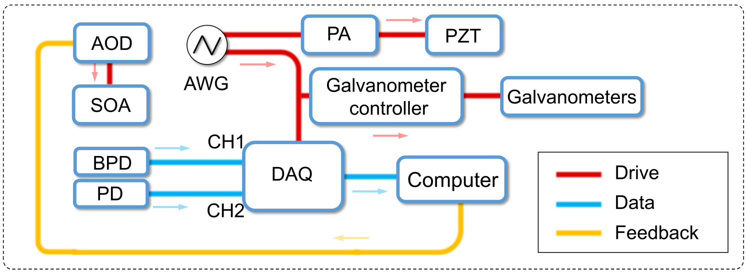

Fig. S3 Schematic diagram of signal generation and acquisition in 3D imaging. AWG, arbitrary waveform generator. DAQ, data acquisition card. PZT, piezoelectric ceramic actuator. PA, power amplifier. PD, photodetector. BPD, balanced photodetector. AOD, analog output device. SOA, semiconductor optical amplifier. The red, blue, and yellow lines represent the drive signals, data signals, and feedback control signals.

4 Performance of adaptive intensity compensation

Higher-order harmonics exhibit significant sensitivity to optical feedback strength. To address this issue, we implement an adaptive compensation scheme using optical circulators and an SOA in the configuration illustrated in Fig. S4a. We first calculate the SNR of the fundamental signal, using a 60 dB threshold, and then dynamically adjust the drive current to regulate the measurement light intensity, thereby stabilizing the feedback strength. Increasing the SNR threshold can support higher-order harmonics.

A comparison of the SNR of the 1st to 3rd harmonic signals before and after compensation in 3D imaging experiments is presented in Fig. S4b and c, where median SNR values are used to characterize the intensity response. The 3rd harmonic exhibits over 42 dB enhancement with compensation. Without compensation, 41% of the 3rd harmonic data points fall below 10 dB, which is reduced to 1% after compensation. Additionally, slight SNR discrepancies between upward and downward scans under identical conditions are observed, primarily attributable to polarization matching variations.

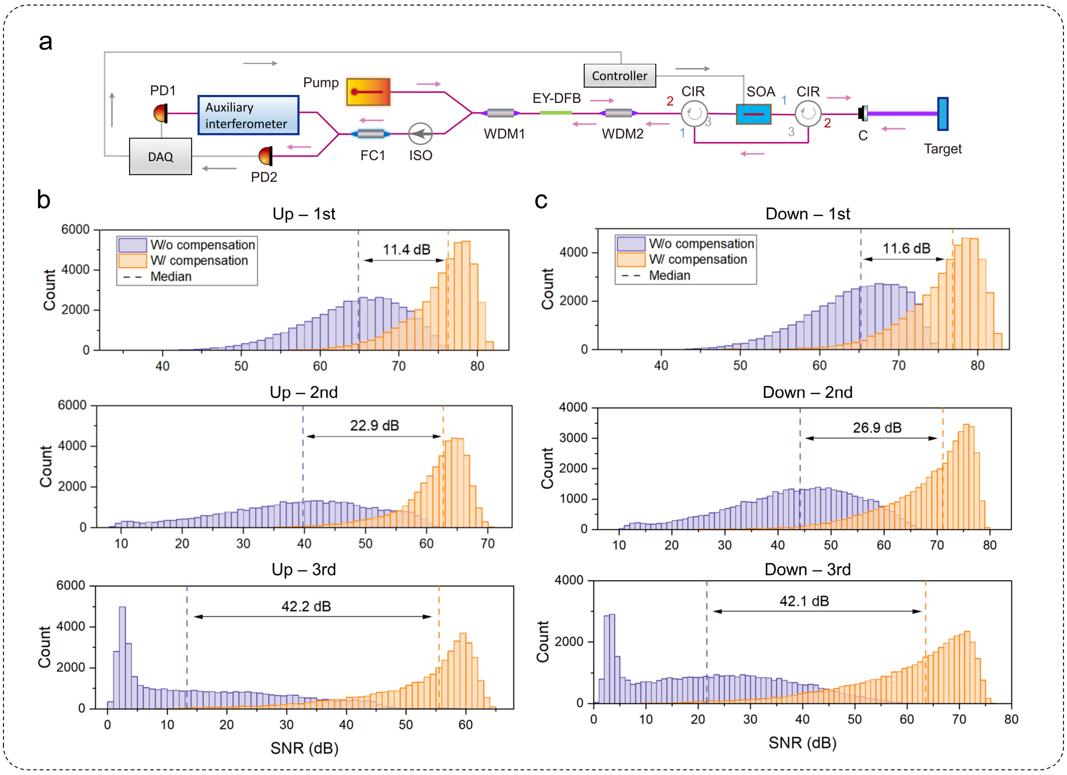

Fig. S4 Adaptive intensity compensation device. a Schematic diagram of the experimental setup. The controller connects the external modulation port of the SOA, and the gain or loss factor is determined by the output voltage. CIR, circulator. **b** The histograms of the SNR for harmonics from the 1st to 3rd in upward scanning before and after compensation. The data originates from 3D-imaging results in Fig. 4b. The purple and orange dashed lines denote the median SNR with and without compensation. **c** The histograms of the SNR for harmonics from the 1st to 3rd in downward scanning.

5 Performance in long-distance measurement

To validate the long-distance measurement capability, we conduct an additional experiment. The target is placed at an optical path length of 104 m, with a feedback power attenuation of approximately 5×10^{-8}. As shown in Fig. S5a, the 4th harmonic still maintains an SNR of nearly 20 dB. Results from 10 repeated measurements yield a standard deviation of around 80.6 μm (Fig. S5b), which is consistent with the performance observed in short-range tests (Fig. 3g). This confirms that the system remains functional at extended ranges.

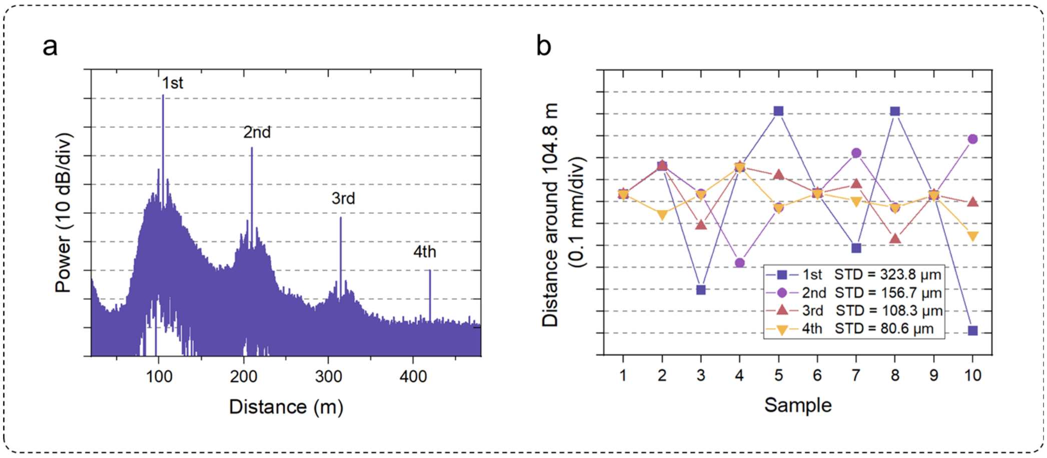

Fig. S5 Ranging results for a target at 104 m. a Power spectrum of the ranging results. The SNR of the 4th harmonic is nearly 20 dB, with 5×10^{-8} feedback power attenuation. **b** Repeated ranging results of the beat signal and its harmonics from the 2nd to the 4th. STD, standard deviation.

6 Comparison with the Conventional FMCW Ranging System

Comparison experiments are conducted to evaluate the enhancement in SNR for both the harmonics and the fundamental interference signal induced by laser feedback. The experimental setups for the two systems, the conventional FMCW and the LFPM ranging system, are shown in Fig. S6. Identical experimental conditions are maintained, including laser output power, target distance and orientation, and beam divergence angle, with all components shared except for some devices marked in the figure. In the conventional system, an optical circulator, CIR1, is used for transmitting and collecting the measurement beam, and two fiber couplers, FC2 and FC3, generate the interference signal. The power emitted from the collimator is 41 μW, and PD1 receives 24 μW of the combined beam. In contrast, for the LFPM system, interference occurs intrinsically within the laser cavity, eliminating the external reference arm. Consequently, the output beam is directed onto the target, and the interference signal is acquired by directly monitoring the laser output. An adjustable optical attenuator, ATT1, is employed to adjust the measurement beam power to 21 μW, approximately half that of the conventional system. Additionally, another attenuator, ATT2, is used to control the power incident on PD2, matching the level at PD1. The comparison results are detailed in the main text.

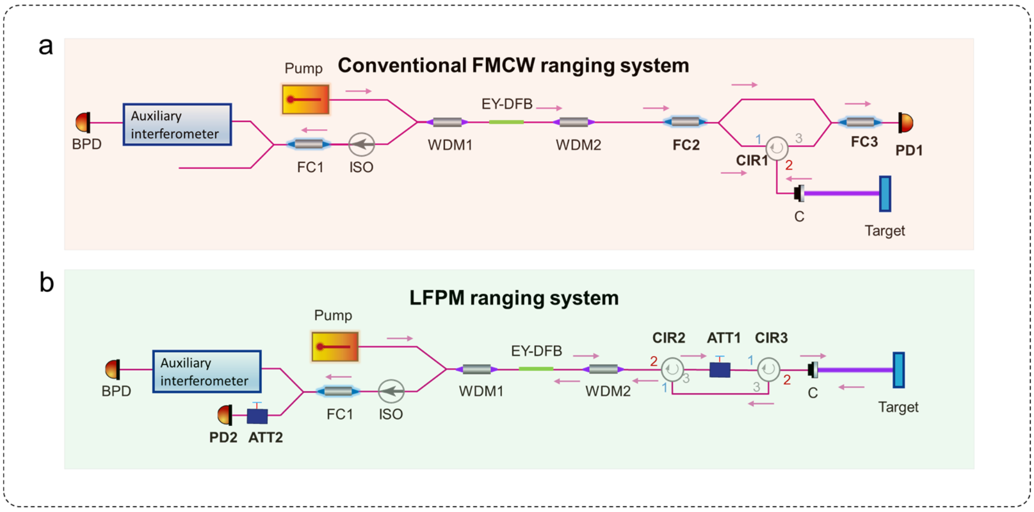

Fig. S6 Schematics of experimental setups. a Conventional FMCW ranging system. **b** The LFPM ranging system. All components are shared except those highlighted in bold.

7 Response frequency bandwidths for harmonics

The response bandwidth of the LFPM system is fundamentally limited by the cavity dynamics. A key metric for quantitatively characterizing cavity dynamics is the RO frequency, which is determined by the parameters of the gain medium, pump source, and laser cavity[3-5]. The LFPM system yields the most significant enhancement, manifested as the strongest intensity and phase modulation, for beat signals at frequencies matching the RO frequency.

Notably, the LFPM system remains operational even when the beat frequency exceeds the RO peak, albeit with a reduced gain. Our system demonstrates the capability to respond to modulation frequencies tens of times higher than the RO peak frequency. For a convincing demonstration, we have performed additional experiments based on the frequency-shift configuration, as depicted in Fig. S7a. First, we utilize AOMs to generate a 6 MHz frequency shift (the maximum supported by our modulator). The spectrum in Fig. S7b clearly reveals the excitation of harmonics up to the 5th order. Considering that the RO frequency is approximately 0.56 MHz, this result indicates that the system's response bandwidth for the fundamental beat signal reaches nearly 11 times the RO frequency, while the bandwidth for harmonics exceeds 50 times the RO frequency. Additionally, the experimental setup for swept-frequency modulation is shown in Fig. S7c, with a target distance set at 170.8 m. The results in Fig. S7d demonstrate the excitation of the 3rd harmonic. The equivalent distance corresponding to the RO peak is about 6.8 m, which indicates that the response bandwidth extends to 25 times for the fundamental beat signal and up to 75 times for the harmonics.

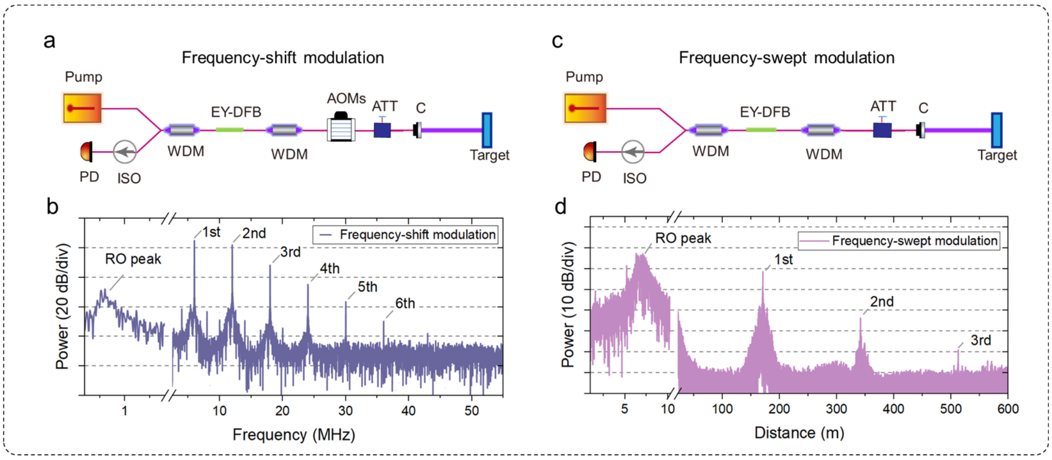

Fig. S7 System response at beat frequencies exceeding the RO peak. a Experimental setup with frequency-shift modulation. **b** The power spectrum of laser output. **c** Experimental setup with frequency-swept modulation. **d** The ranging result.

Nevertheless, as the modulation frequency increases further (e.g., to hundreds of times the RO frequency), higher-order harmonics become difficult to excite, making resolution enhancement unattainable. Thus, the cavity dynamics ultimately determine the upper limit of the usable modulation bandwidth.

8 Ranging results with CZT

We further evaluate the precision of distance measurements derived from different harmonics. In contrast to the tests in Fig. 3, the data here are processed using the chirp-Z transform (CZT) for refined spectral interpolation. It is worth noting that while CZT or zero-padding interpolation can improve the data resolution to facilitate the extraction of the peak frequency of the signal, they cannot reduce the intrinsic spectral bandwidth of the signal[6]. In the experiment, an optical fiber mirror is used as the target, located approximately 3.13 m from the light source. The output power of the laser source is 1.7 mW, with a maximum sweep bandwidth of 110 GHz. By applying CZT, the frequency sampling interval is refined, and the data resolution is 0.1 μm. The measurement results are shown in Fig. S8a, where the inset presents the detailed spectra of the 1st and 13th harmonics. Across 10 repeated measurements, the standard deviations of the 9th-order and lower harmonics are close to each other and remain below 3 μm.

The linearity test of the system is also carried out, which demonstrates the nonlinear error in measurements. The precision of the stage is 50 nm, which can be taken as the standard. An aluminum sheet serves as the target, which is driven by the stage within 10 cm, covering the whole travel range of the stage. The acquired data, linear fitting, and residual error are shown in Fig. S8b. The maximum residual error is 43 μm from the 4th harmonics, corresponding to a linearity of 4.3×10^{-4} within 10 cm.

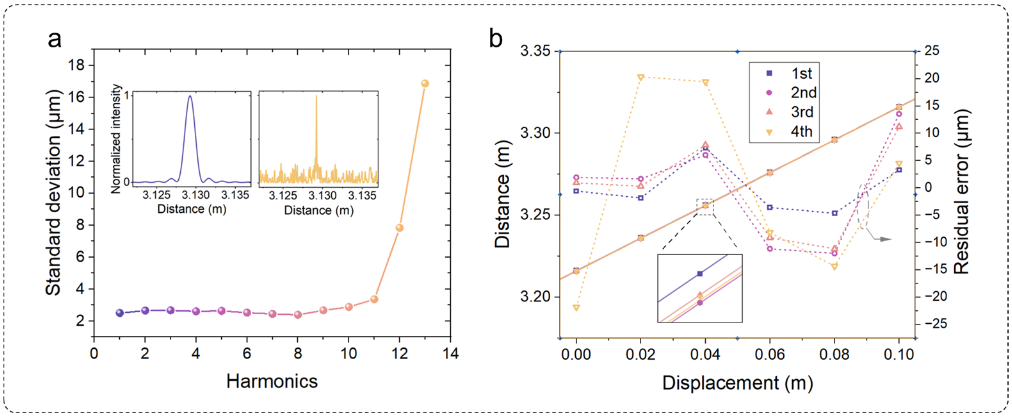

Fig. S8 Ranging results of harmonics using CZT. a Precision test of harmonics. We employ a reflector as the target, and repeat 10 measurements. The standard deviations of the 1st to 13th harmonics are shown. The insets show the local spectra of the 1st and 13th with normalized intensity, highlighting the clear difference in FWHMs. **b** Linearity test of harmonics. Solid lines denote the linear-fitting results, and dashed lines represent the residual error of ranging employing the 1st to 4th harmonics. The inset shows the details of the data at 0.04 m.

9 Solutions to the operating frequency equation

The evolution of the laser under the influence of frequency-shift feedback is governed by Eq. (4) in the main text[7]. This equation can be analyzed graphically by defining the left-hand side as $f_1(\omega)$ and the right-hand side as $f_2(\omega)$, and plotting them in the same coordinate system.

$$f_1(\omega) = \omega(t) - \omega_c$$
$$f_2(\omega) = \kappa\gamma_c \sin[\Omega t - \omega(t)\tau] \tag{S1}$$

As shown in Fig. S9a, the two curves intersect at least once. The number of solutions depends critically on the feedback strength κ, and can be categorized into three regimes: 1) $\kappa\gamma_c\tau < 1$, the magnitude of the derivative $|f_2'(\omega)|$ is less than 1, and the equation has only one solution, corresponding to single-mode operation of the laser; 2) $\kappa\gamma_c\tau = 1$, the critical condition of single-mode operation; 3) $\kappa\gamma_c\tau > 1$, multiple modes are excited, leading to mode hopping within the laser resonator. Notably, all experimental investigations in this study are conducted under single-mode operation to ensure the validity of the measurements.

Additionally, the frequency difference Ω between the feedback light and the intracavity light introduces a time-varying phase shift, even if the feedback strength remains constant over time. The solutions under different instantaneous phase shifts, as depicted in Fig. S9b, reveal that the actual output frequency of the laser will oscillate periodically within a bounded range, with the size of this range proportional to the feedback strength κ.

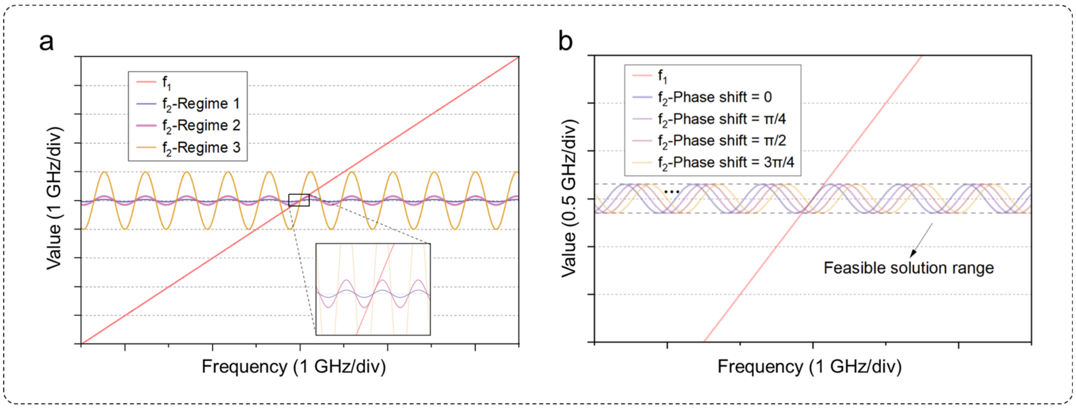

Fig. S9 Graphical analysis of the operating frequency equation solutions. a Define two functions, f_1 and f_2, representing the expression of the equation, and their intersections denote the solution. The inset shows the details of the intersection. According to the feedback strength, the solution can be classified into three regimes, corresponding to $\kappa\gamma_c\tau$ 1) less than, 2) equal to, or 3) larger than 1. **b** Solutions of the equation with various phase shifts in regime 1) or 2). The dashed line represents the feasible range of solutions.

10 Linewidth analysis

The linewidth of the free-running laser is measured using the delayed self-heterodyne technique. The experimental configuration, illustrated in Fig. S10a, employs a fiber coupler to split the output of the distributed feedback (DFB) fiber laser into two paths. One path propagates through AOMs applying a 6 MHz frequency shift, while the other is delayed by a 30 km single-mode fiber (SMF). The combined optical signal is detected by a PD and analyzed using an ESA. The power spectrum, referenced to the AOM shift, is presented in Fig. S10b. Owing to the influence of the noise near the center frequency, the -3 dB linewidth often overestimates the actual Lorentzian linewidth. Therefore, we estimate the intrinsic linewidth from the -20 dB bandwidth, which equals $2\sqrt{99}$ times the intrinsic Lorentzian linewidth. The calculated linewidth is 26.4 kHz, corresponding to a coherence length greater than 11.4 km.

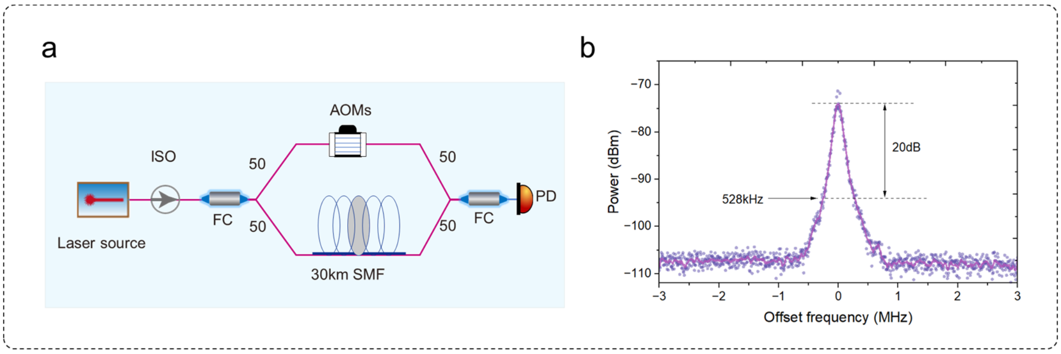

Fig. S10 Linewidth characterization of the employed DFB fiber laser. a Schematic diagram of the delayed self-heterodyne interferometer for coherence length evaluation**.** SMF, single-mode fiber. The isolator is used to block the back-scattering beams, which may induce laser feedback. **b** The frequency-offset spectrum of the detected signal. The linewidth at -20 dB is 528 kHz, corresponding to the linewidth of 26.4 kHz.

11 Fabrication of the swept laser source

The DFB fiber laser is selected as the light source, and the resonator cavity is approximately 50 mm in length. To ensure continuous frequency sweeping, the optical fiber resonator is maintained under constant tensile strain through the application of a preload stress, which is precisely adjusted by monitoring the lasing wavelength. The pre-stressed resonator is then affixed to a PZT using adhesive until complete curing. A 72-mm PZT element is employed to match the resonator length, and it is driven by a symmetric triangular wave signal amplified through a power amplifier.

As illustrated in Fig. S11, the frequency-swept bandwidth at 10 Hz, 40 Hz, and 100 Hz modulation frequencies exhibits near-identical magnitudes, achieving a 110 GHz sweep bandwidth under 100 Vpp drive voltage. Experimental operations utilize 100 Vpp at 80 Hz.

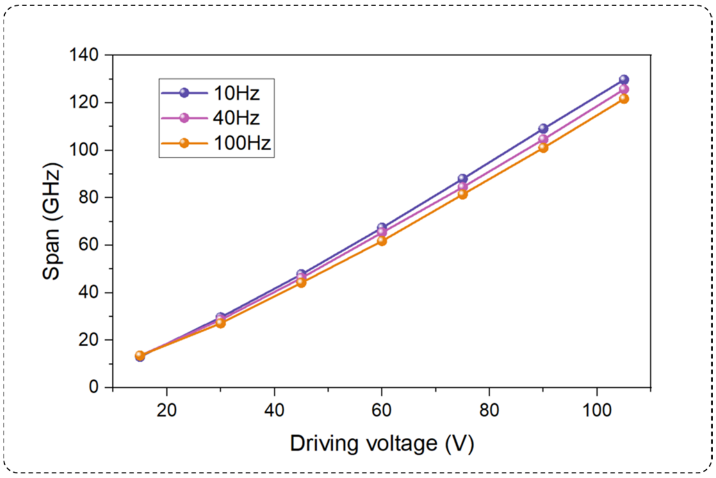

Fig. S11 Frequency-swept bandwidth as a function of drive voltage amplitude at different modulation frequencies.

12 Modulation repeatability via PZT

In this work, the laser cavity is modulated by mechanically stretching a fiber laser using a PZT. Although fluctuations in PZT performance during modulation may affect certain laser output characteristics, their impact on the final ranging results is negligible. Below, we provide a detailed analysis of both wavelength and power.

The stability of the PZT motion directly affects both the wavelength repeatability and the sweep nonlinearity. However, our ranging method, similar to the FMCW ranging, is inherently insensitive to the absolute values of the start and stop wavelengths. To quantify the sweep stability, we conduct 100 repeated measurements of the sweep bandwidth. As shown in Fig. S12a, the standard deviation of the sweep bandwidth is 13.8 MHz, which corresponds to a relative variation of 1.2×10^{-4}. This minor fluctuation has a negligible effect on the ranging resolution. Furthermore, we evaluate the consistency of the phase increments in auxiliary signals during frequency sweeps. The results from five repeated tests show nearly overlapping curves, as depicted in Fig. S12b. The inset reveals only minor phase deviations on the order of tens of radians. Importantly, these deviations vary slowly over time and represent sweep nonlinearities that can be effectively compensated using resampling methods.

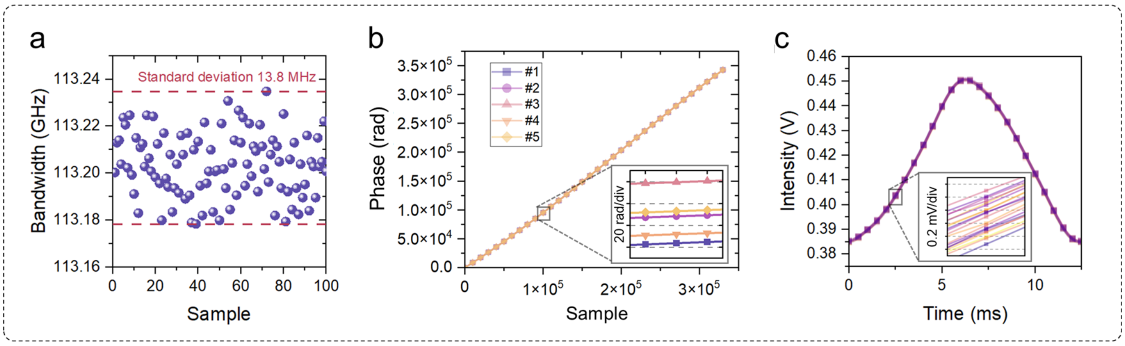

Fig. S12 Evaluations of modulation repeatability via PZT. a Repeatability of the sweep bandwidth during 100 measurements. **b** Phase increments of the auxiliary signal in 5-times repeated sweep modulation. The phase deviations represent the repeatability of the modulation, and the inset shows the details. **c** Temporal intensity fluctuations detected by a PD, which are proportional to laser output power. The inset shows the results in 20 periods.

Additionally, mechanical cavity-length tuning via the PZT can modulate the output power of the laser. We record the laser output intensity in single modulation 20 times using a PD, and the results are presented in Fig. S12c. The measurement beam intensity varies between 0.385 V and 0.45 V. The detailed deviation is shown in the inset, where the relative deviation is 2.3×10^{-3}. In the LFPM system, the critical parameter for ranging is the peak frequency of the harmonic signal. Power fluctuations hardly influence the detection of this peak frequency, provided they occur at rates much slower than the beat frequency. For a target at 10 meters,

the beat signal frequency is on the order of MHz, far exceeding the frequency of power variations. Therefore, these slow fluctuations have minimal effect on the accuracy of peak frequency identification and the subsequent distance measurement.

13 Feasibility of resampling in the LFPM system

Current studies indicate that the k-sampling calibration method requires satisfaction of the condition[8-10]:

$$|\tau_a^2 \alpha| \ll 1 \tag{S2}$$

where τ_a denotes the auxiliary path delay, $\alpha = d\nu/dt$ represents the optical frequency sweep rate, ν is the optical frequency, and t is time.

Here, we rigorously analyze the validity of this original condition under swept-frequency feedback with increasing feedback strength κ, and derive a revised stability criterion. From Eq. (4) in the main text, the optical frequency evolution in the swept-frequency feedback system is given by[11,12]:

$$\omega(t) = \omega_{c0} + 2\pi\alpha_0 t + \gamma_{ext} \sin\left[\int_{t-\tau_m}^{t} \omega(x)dx\right] \tag{S3}$$

where $\gamma_{ext} = \kappa\gamma_c$, ω_{c0} is the initial laser local oscillation frequency, α_0 is the sweep rate without feedback, and τ_m is the measurement path delay. This derivation assumes weak feedback conditions. When $\gamma_{ext}\tau_m \le 1$, the system exhibits a single solution (mode-hop-free operation), preserving phase continuity for FMCW ranging. Under this condition, the frequency sweep rate α_0 can be treated as a constant.

Differentiating Eq. (S3) with respect to t yields the actual sweep rate under feedback $\alpha_f(t)$:

$$2\pi\alpha_f(t) = \frac{d\omega(t)}{dt} = 2\pi\alpha_0 + \gamma_{ext} \cos\left[\int_{t-\tau_m}^{t} \omega(x)dx\right][\omega(t) - \omega(t-\tau_m)] \tag{S4}$$

Expanding $\omega(t-\tau_m)$ as a Taylor series around t:

$$\omega(t-\tau_m) = \omega(t) - \frac{d\omega(t)}{dt}\tau_m + \frac{d^2\omega(t)}{2dt^2}\tau_m^2 + \cdots \tag{S5}$$

Assuming the second- and higher-order terms are negligible, which means

$$|\alpha_f(t)| \gg \left|\frac{d\alpha_f(t)}{2dt}\tau_m\right| \tag{S6}$$

We obtain the linearized equation for $\alpha_f(t)$:

$$2\pi\alpha_f(t) = 2\pi\alpha_0 + \gamma_{ext} \cos\left[\int_{t-\tau_m}^{t} \omega(x)dx\right] 2\pi\alpha_f(t)\tau_m \tag{S7}$$

Solving for the actual sweep rate $\alpha_f(t)$ gives:

$$\alpha_{\mathrm{f}}(t)=\frac{\alpha_0}{1-\gamma_{\mathrm{ext}}\tau_{\mathrm{m}}\cos\left[\int_{t-\tau_{\mathrm{m}}}^{t}\omega(x)dx\right]} \tag{S8}$$

Therefore, the resampling criterion becomes:

$$\max(|\alpha_{\mathrm{f}}(t)\tau_{\mathrm{a}}^2|)=\max\left(\left|\frac{\alpha_0}{1-\gamma_{\mathrm{ext}}\tau_{\mathrm{m}}\cos\left[\int_{t-\tau_{\mathrm{m}}}^{t}\omega(x)dx\right]}\tau_{\mathrm{a}}^2\right|\right)\ll 1 \tag{S9}$$

which simplifies to:

$$\frac{\alpha_0\tau_{\mathrm{a}}^2}{1-\gamma_{\mathrm{ext}}\tau_{\mathrm{m}}}\ll 1 \tag{S10}$$

The following equations are derived to validate the assumption that the second-order quantity is much smaller than that of the first-order. Refer to Eq. (S6), and take the derivative of $\alpha_{\mathrm{f}}(t)$ with respect to t and ignore higher-order infinitesimals:

$$\frac{d\alpha_{\mathrm{f}}(t)}{2dt}\frac{\tau_{\mathrm{m}}}{\alpha_{\mathrm{f}}(t)}=-\frac{\gamma_{\mathrm{ext}}\tau_{\mathrm{m}}^3\alpha_{\mathrm{f}}^2(t)/\alpha_0}{1+\gamma_{\mathrm{ext}}\tau_{\mathrm{m}}^3\alpha_{\mathrm{f}}^2(t)/\alpha_0\cdot\sin\left[\int_{t-\tau_{\mathrm{m}}}^{t}\omega(x)dx\right]}\sin\left[\int_{t-\tau_{\mathrm{m}}}^{t}\omega(x)dx\right] \tag{S11}$$

For resampling based on auxiliary interference at π-phase interval, the Nyquist theorem requires $\tau_{\mathrm{m}}<2\tau_{\mathrm{a}}$. When $\alpha_0\tau_{\mathrm{a}}^2/(1-\gamma_{\mathrm{ext}}\tau_{\mathrm{m}})\ll 1$, it follows that $\alpha_0\tau_{\mathrm{m}}^2/(1-\gamma_{\mathrm{ext}}\tau_{\mathrm{m}})\ll 1$. The further analysis can be classified into two regimes:

1) For cases of weak feedback, where $\gamma_{\mathrm{ext}}\tau_{\mathrm{m}}\ll 1$, α_{f} and α_0 are comparable. Thus, $\gamma_{\mathrm{ext}}\tau_{\mathrm{m}}^3\alpha_{\mathrm{f}}^2(t)/\alpha_0\ll 1$, leading to $|\alpha_{\mathrm{f}}(t)|\gg|\frac{d\alpha_{\mathrm{f}}(t)}{2dt}\tau_{\mathrm{m}}|$.
2) For cases of moderate feedback, where $\gamma_{ext}\tau_{\mathrm{m}}$ is comparable to 1, the condition $|\alpha_{\mathrm{f}}(t)|\gg\left|\frac{d\alpha_{\mathrm{f}}(t)}{2dt}\tau_{\mathrm{m}}\right|$ must be satisfied as follows:

$$\max\left\{\gamma_{\mathrm{ext}}\tau_{\mathrm{m}}^3\alpha_{\mathrm{f}}^2(t)/\alpha_0\cdot\sin\left[\int_{t-\tau_{\mathrm{m}}}^{t}\omega(x)dx\right]\right\}\approx\max\left[\frac{\tau_{\mathrm{m}}^2\alpha_{\mathrm{f}}^2(t)}{\alpha_0}\right]\ll 1 \tag{S12}$$

Further simplification shows that:

$$\frac{\alpha_0\tau_{\mathrm{m}}^2}{(1-\gamma_{\mathrm{ext}}\tau_{\mathrm{m}})^2}\ll 1 \tag{S13}$$

In this work, $\kappa\gamma_{\mathrm{c}}$ operates lower than 1 MHz, with the measurement distance on the meter scale. The corresponding round-trip time τ_{m} and τ_{a} are on the order of 50 ns. The chirp rate of the system is around 10 THz s^{-1}, which satisfies the above criteria for implementing resampling to suppress sweep nonlinearity.

14 Potential crosstalk between the ranging signal and the auxiliary signal

The auxiliary interferometer shares the same laser source as the LFPM ranging system, and the raw auxiliary signal contains frequency components of the ranging beat and its harmonics. However, according to the operating principle of resampling techniques, the optical path delay (L_{aux}) in the auxiliary interferometer is designed to be several times longer than the stand-off distance of the ranging measurement. Consequently, the frequency of the auxiliary interference fringes is significantly higher than that of the primary ranging signals. Even in scenarios where they might theoretically overlap, such as when high-order sidebands are excited and the delay line of the auxiliary interferometer is relatively short, the power of these high-order sidebands is substantially lower than that of the dominant auxiliary interference signal. Therefore, their impact on the resampling process and the subsequent nonlinearity compensation is negligible.

In our specific system configuration, L_{aux} exceeds 140 m, which is more than ten times the typical target distance under test. This design provides clear spectral separation between the two signals. Accordingly, a high-pass filter can be applied to the auxiliary signal to effectively eliminate any potential influence from the ranging signal components. The power spectra of both the raw auxiliary signal and the filtered signal are presented in Fig. S13. Additionally, an optical isolator is incorporated into the auxiliary interferometer setup. This serves to suppress backscattered light originating from optical components of the auxiliary interferometer or the PDs. Consequently, the disturbances can be mitigated, ensuring the stable operation of the entire system.

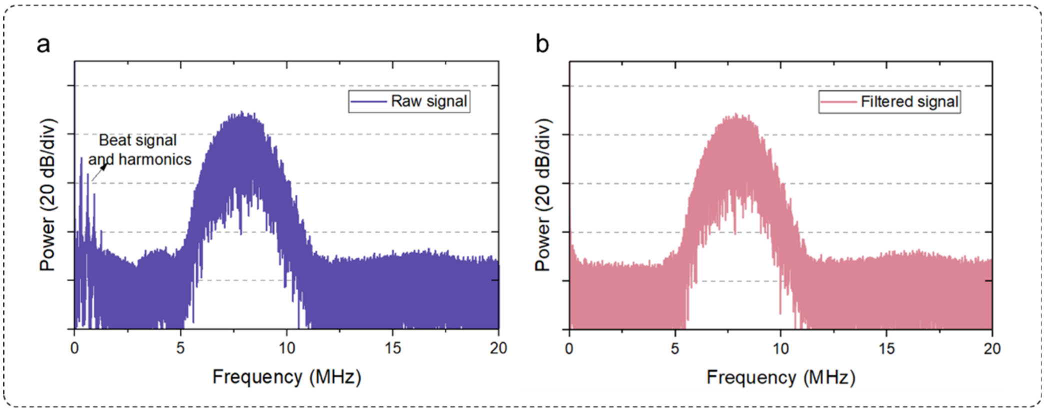

Fig. S13 Power spectra of the auxiliary signal. a Raw auxiliary interference signal. It contains the components of the ranging beat signal and its harmonics. **b** The high-pass-filtered auxiliary interference signal.

References

1 Tan, Y. D., Zhang, S. L., Zhang, S., Zhang, Y. Q. & Liu, N. Response of microchip solid-state laser to external frequency-shifted feedback and its applications. *Scientific Reports* **3**, 2912 (2013).

2 Wang, Y. F. *et al.* Frequency-swept feedback interferometry for noncooperative-target ranging with a stand-off distance of several hundred meters. *Photonix* **3**, 21 (2022).

3 Li, J. *et al.* Toward exploring noncontinuous-state dynamics based on pulse-modulated frequency-shifted laser feedback interferometry. *Photonics Research* **13**, 671-686 (2025).

4 Zenteno, L. A., Snitzer, E., Po, H., Tumminelli, R. & Hakimi, F. Gain Switching of a Nd-+3-Doped Fiber Laser. *Optics Letters* **14**, 671-673 (1989).

5 Hou, Y. B., Zhang, Q., Qi, S. X., Feng, X. & Wang, P. Monolithic all-fiber repetition-rate tunable gain-switched single-frequency Yb-doped fiber laser. *Optics Express* **24**, 28762-28768 (2016).

6 Hao, Y., Song, P., Wang, X. & Pan, Z. A Spectrum Correction Algorithm Based on Beat Signal of FMCW Laser Ranging System. *Sensors* **21**, 5057 (2021).

7 Zhang, S., Zhang, S., Sun, L. & Tan, Y. Spectrum Broadening in Optical Frequency-Shifted Feedback of Microchip Laser. *IEEE Photonics Technology Letters* **28**, 1593-1596 (2016).

8 Zehao, Y., Cheng, L. & Guodong, L. FMCW LiDAR with an FM nonlinear kernel function for dynamic-distance measurement. *Optics Express* **30**, 19582-19596 (2022).

9 Moore, E. D. & McLeod, R. R. Correction of sampling errors due to laser tuning rate fluctuations in swept-wavelength interferometry. *Optics Express* **16**, 13139-13149 (2008).

10 Barber, Z. W., Babbitt, W. R., Kaylor, B., Reibel, R. R. & Roos, P. A. Accuracy of active chirp linearization for broadband frequency modulated continuous wave ladar. *Applied Optics* **49**, 213-219 (2010).

11 Taimre, T. *et al.* Laser feedback interferometry: a tutorial on the self-mixing effect for coherent sensing. *Advances in Optics and Photonics* **7**, 570-631 (2015).

12 Lacot, E., Day, R. & Stoeckel, F. Laser optical feedback tomography. *Optics Letters* **24**, 744-746 (1999).
